# NPBSS: a new PacBio sequencing simulator for generating the continuous long reads with an empirical model

**DOI:** 10.1186/s12859-018-2208-0

**Published:** 2018-05-22

**Authors:** Ze-Gang Wei, Shao-Wu Zhang

**Affiliations:** Key Laboratory of Information Fusion Technology of Ministry of Education, School of Automation, Northwestern Polytechnical University, Xi’an, 710072 China

**Keywords:** Sequence simulator, Quality value, Continuous long reads, SMRT, PacBio

## Abstract

**Background:**

PacBio sequencing platform offers longer read lengths than the second-generation sequencing technologies. It has revolutionized de novo genome assembly and enabled the automated reconstruction of reference-quality genomes. Due to its extremely wide range of application areas, fast sequencing simulation systems with high fidelity are in great demand to facilitate the development and comparison of subsequent analysis tools. Although there are several available simulators (e.g., PBSIM, SimLoRD and FASTQSim) that target the specific generation of PacBio libraries, the error rate of simulated sequences is not well matched to the quality value of raw PacBio datasets, especially for PacBio’s continuous long reads (CLR).

**Results:**

By analyzing the characteristic features of CLR data from PacBio SMRT (single molecule real time) sequencing, we developed a **n**ew **P**ac**B**io **s**equencing **s**imulator (called NPBSS) for producing CLR reads. NPBSS simulator firstly samples the read sequences according to the read length logarithmic normal distribution, and choses different base quality values with different proportions. Then, NPBSS computes the overall error probability of each base in the read sequence with an empirical model, and calculates the deletion, substitution and insertion probabilities with the overall error probability to generate the PacBio CLR reads. Alignment results demonstrate that NPBSS fits the error rate of the PacBio CLR reads better than PBSIM and FASTQSim. In addition, the assembly results also show that simulated sequences of NPBSS are more like real PacBio CLR data.

**Conclusion:**

NPBSS simulator is convenient to use with efficient computation and flexible parameters setting. Its generating PacBio CLR reads are more like real PacBio datasets.

**Electronic supplementary material:**

The online version of this article (10.1186/s12859-018-2208-0) contains supplementary material, which is available to authorized users.

## Background

The single molecule real-time (SMRT) sequencing, developed by Pacific Biosciences (PacBio), is a newly emerging third-generation DNA sequencing technology [1]. PacBio’s SMRT sequencing is also the first commercially available long-read sequencing technology currently in use [2, 3]. Compared with second generation sequencing (also called high-throughput sequencing), such as Illumina [4], Roche 454 [5] and SOLiD [6], the PacBio sequencing system is significantly less expensive per run, does not rely on amplification for library generation, and supports shorter turn-around time [7]. PacBio produces two types of reads. One is the continuous long reads (CLR) with an average error rate of ~ 15%, and the other one is the circular consensus sequencing (CCS) short reads with high accuracy of > 97% from multiple passes across insert sequences [3]. The requirement that three or more full passes across insert sequences for CCS reads limits the insert size to < 2.5 kb, but the CLR reads can rang up to ~ 40 kb by using a DNA polymerase anchored in a zero-mode waveguides [4, 8–10]. In contrast, the second generation sequencers typically generate much shorter reads with median lengths of ~ 100–250 bp for Illumina and ~ 500 bp for Roche 454 [11, 12]. Therefore, the CLR reads generated by the PacBio platform is a key progression in the high-throughput sequencing technologies, which is expected to benefit many aspects of genomic projects in near future [13–15]. The long sequence can span extended repetitive regions and thereby have more power to reveal complex structural variations presenting in the DNA samples, such as pinpointing precisely where copy number variations occur relative to the reference sequence [16]. The de novo genome assembly will also benefit from PacBio sequencing because long reads can provide large scaffolds, and it is becoming routine for bacterial genomes to be completely assembled using PacBio sequencing platform [17].

So far, many computational methods and efficient software tools have been developed to process sequences produced by PacBio. Generally, these methods need to be benchmarked using simulated data. Because the simulated data can be generated as much similar as desired and under controlled situations with predefined parameters [18]. In addition, it’s also low-cost and time efficient to generate simulation datasets [19, 20]. As a result, the genome sequencing simulators have become increasingly popular for assessing and validating computational methods or for gaining an understanding of specific data sets [18]. Sequence simulators can be applied to help develop and evaluate downstream analysis tools, such as the correctness of an assembly [21], the accuracy of gene prediction [22] and sequence clustering [23, 24], or the power to reconstruct accurate genotypes and haplotypes [25]. Therefore, sequence simulators will benefit for many relevant bioinformatics applications.

There are several read simulators targeted to generate the PacBio reads, such as, SimLoRD [26], PBSIM [27] and FASTQSim [28]. SimLoRD [26] software is specially designed for PacBio CCS reads generation. It offers the options of choosing the read length distribution and modelling the error probabilities depending on the number of passes through the sequencer. Therefore, SimLoRD is more convenient than PBSIM and FASTQSim for parameters setting to simulate PacBio CCS reads. PBSIM [27] and FASTQSim [28] can simulate CCS reads and CLR reads. Although PBSIM [27] simulates PacBio reads well, there are the following two limitations. First, the quality value (QV, also called the Phred quality score) at each position for a simulated read is randomly chosen, but we found that the proportions of different QVs in real PacBio reads are different (see Methods for details). Second, we also observed that the error rate of simulated reads is higher than QV (see Methods for details). FASTQSim [28] provides both read analysis and simulation for the second generation and PacBio sequencing platforms. By characterizing the error profiles from datasets provided by users, FASTQSim generates the simulated reads. However, FASTQSim takes long time in simulating, and it is not flexibly to directly change parameters. In addition, the error rate of simulated sequences produced by PBSIM and FASTQSim is not well matched to the QV [26]. It is noteworthy that QV is a measurement of the identification quality of nucleobases. The QV of each base in a sequence reflects the error probability of each position. Therefore, it is crucial to deal with the sequencing errors and QVs for a sequencing simulation tool.

To improve upon the existing solutions, by analyzing some characteristics (i.e., the distribution of sequence length, different types of sequencing errors) on several real datasets generated by the PacBio sequencing platform, and uncovering the relationship between QV and sequencing error rate, we developed a **n**ew **P**ac**B**io **s**equence **s**imulator (called NPBSS) to generate PacBio CLR reads. NPBSS uses an empirical error model derived from the real datasets to simulate different errors for each sequence. Alignment and assembly tests show that the simulated CLR length and quality distributions of NPBSS agree well with the real PacBio data.

## Implementation

**Fig. 1:**
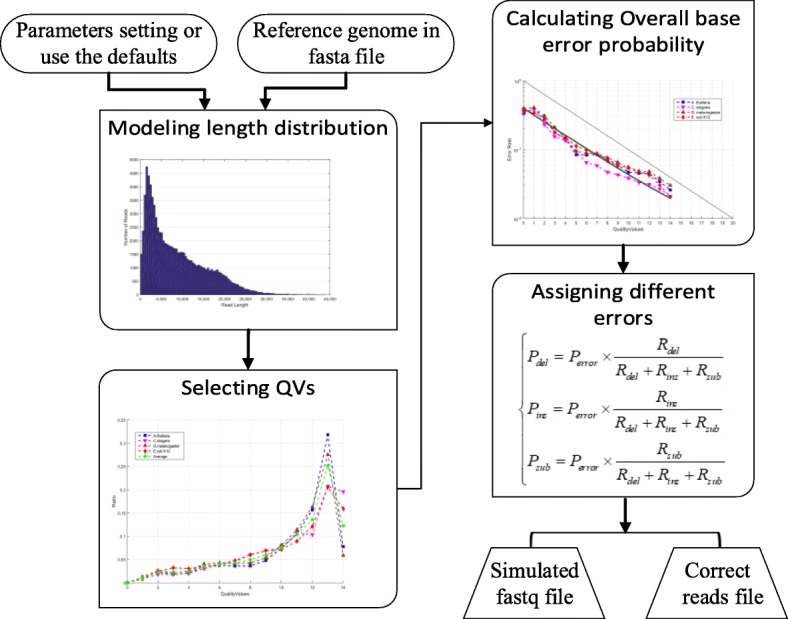
NPBSS simulator workflow

NPBSS was written in MATLAB (a free version of NPBSS under Octave is also available) and has a command line user interface. As shown in Fig. 1, a single run of NPBSS command line consists of four main steps: i) modeling read length distribution, ii) selecting QVs, iii) calculating overall base error probability and iv) assigning different base error probabilities. The required input file, commands and the resulting output files are described below. NPBSS just requires one reference genome input file in FASTA format. The users can adjust the parameters according to their project or directly apply the defaults in NPBSS (see Methods for detail parameter settings). For read length generation, there are four ways: i) providing the mean and standard deviation value for a log-normal distribution (−lg mean std); ii) giving a sequencing depth (−dep); iii) sampling the read length from a FASTA or FASTQ file provided by users (−samp) and iv) offering a sequence number (−n). And users also can set –len (default value: 8500) to determine the value of average read length for –n and –dep options. For QVs selection, NPBSS will choose different QVs from the default QVs table (see Methods), or from the users defined QVs table (−qv table). The default QVs table is recommended to use, because it fits well with the raw PacBio data (see Methods). Based on the empirical model, the base overall error probability is calculated from the QV in each position. For different base error probability assignment, the base error probabilities for reads can be specified individually for substitutions (−sub), insertions (−ins) and deletions (−del). For the following example using the default parameters, where 10,000 reads are simulated, the command line is: NPBSS(‘genome.fa’,’-n 10,000 –sub 0.06 –ins 0.03 –del 0.06′), NPBSS sampled from random positions of the reference ‘genome.fa’, and the different average base error probabilities assigned in simulated reads are 6, 3 and 6% for substitutions, insertions and deletions, respectively (~ 15% base total error probability), the default average length (−len) is 8500. Then, the CLR simulated reads can be found in the ‘npbss_simulated.fq’ file and the correct reads are saved in ‘reads_correct.fa’ file.

## Results and discussion

### Analyses of real PacBio datasets

Four different CLR read datasets and two CCS read datasets sequenced with PacBio’s instrument were used to analyze the hidden features of PacBio long reads. These datasets can be free downloaded from the website links listed in Tables S1-S2 (see Additional file 1). Additional file 1: Tables S3-S6 report some brief statistics of these datasets, and Additional file 1: Figures S1-S2 present the length distribution. To learn how to simulate different errors introduced to reads, we need to analyze real PacBio reads by aligning them to corresponding reference sequences. Here, we adopted the Blast alignment tool [29] to obtain the alignment results and the accuracy. Additional file 1: Figures S3-S5 present the distributions of insertion, deletion and substitution errors, which show a nice uniform layout.

### NPBSS simulator performance

#### Accuracy of NPBSS simulator

In order to evaluate the accuracy of NPBSS simulation, the simulated CLR reads need to be mapped to the reference genomes. For genomes of *E. coli K12*, *C. elegans*, *A. thaliana* and *D. melanogaster*, the NPBSS error parameters were set equally to the error rate of raw CLR data (Additional file 1: Table S5). The error profiles of FASTQSim are characterized by the raw CLR data. In total, 12 CLR datasets were simulated with NPBSS, PBSIM and FASTQSim. Blast alignment tool [29] was utilized to map these simulated reads back to the corresponding genomes with default parameters. The results in Additional file 1: Tables S7-S10 for *E. coli K12*, *C. elegans*, *A. thaliana* and *D. melanogaster* show that the error rate and length of simulated reads with NPBSS, PBSIM and FASTQSim are similar to the raw data, which preliminarily demonstrates that NPBSS could simulate PacBio reads with necessary sequencing errors and fidelity as well.

#### Error rate and quality values

**Fig. 2:**
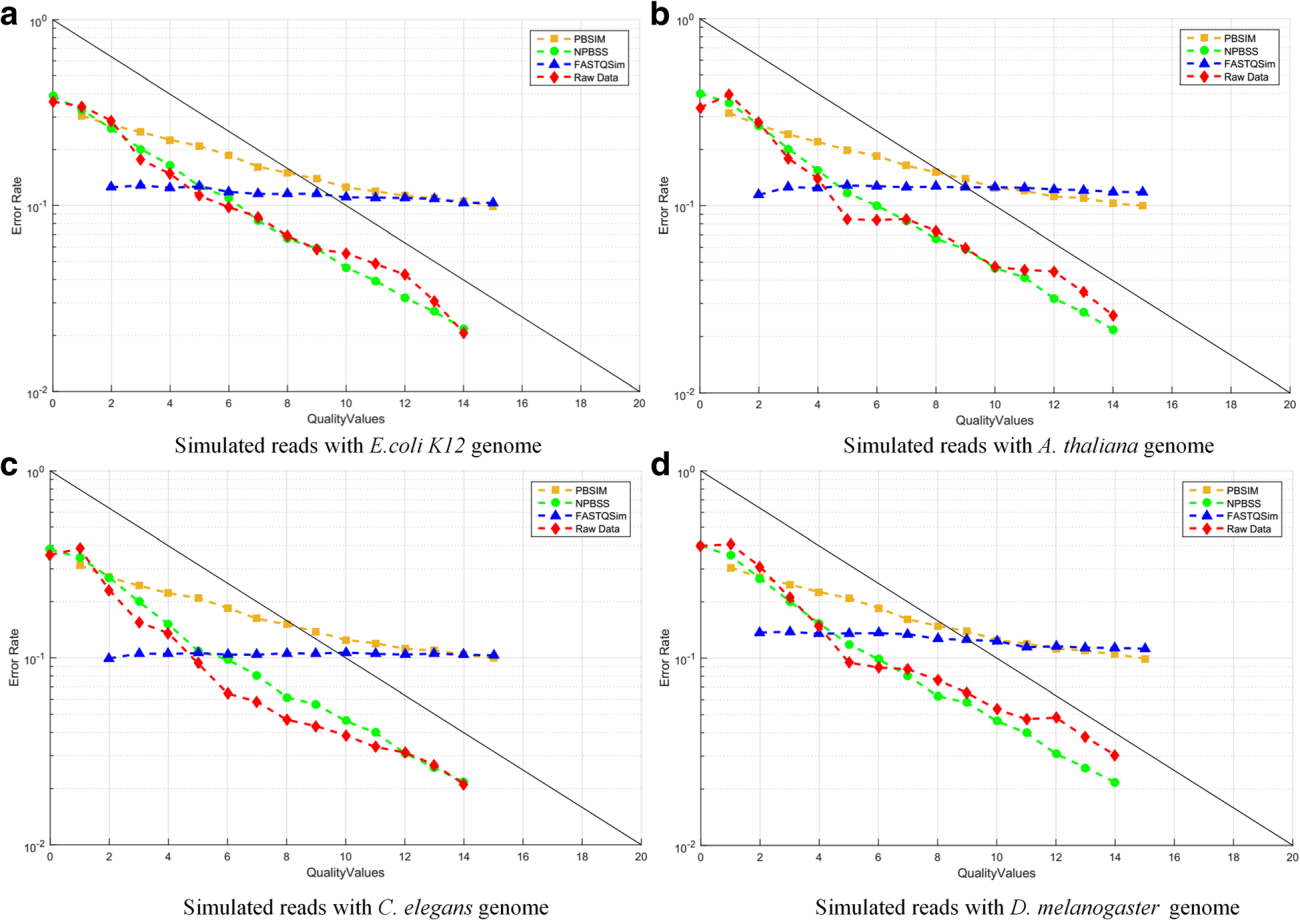
The relationship between error rate and QVs in simulated data generated by NPBSS, PBSIM and FASTQSim for (**a**) *E.coli K12* genome, (**b**) *A. thaliana* genome, (**c**) *C. elegans* genome and (**d**) *D. melanogaster* genome

Next, we want to assess another important fidelity of the sequencing simulators, that is, the relationship between error rate and QVs. We applied above simulated CLR datasets and investigated the error rates. Figure 2 shows the trend between error rate and QVs for NPBSS, PBSIM and FASTQSim. It can be evidently seen that the curve of NPBSS is close to the trend of the four raw data, while PBSIM presents a diverse trend with the growth of QVs, which cannot reflect the true relationship between error rates and QVs. The results of PBSIM can be explained by the fact that the error probability (*P*_*error*_) of a QV in the PBSIM pipeline is directly defined by the QV (${P}_{error}={10}^{-\kern0.5em \frac{QV}{10}}$) values. Although the error profiles are estimated from the raw sequence data, the error rate of simulated reads from FASTQSim does not agree well with the raw data. Therefore, compared with both PBSIM and FASTQSim tools, NPBSS can capture this characterization better and simulate PacBio sequences more reasonably.

#### Computational complexity

**Fig. 3:**
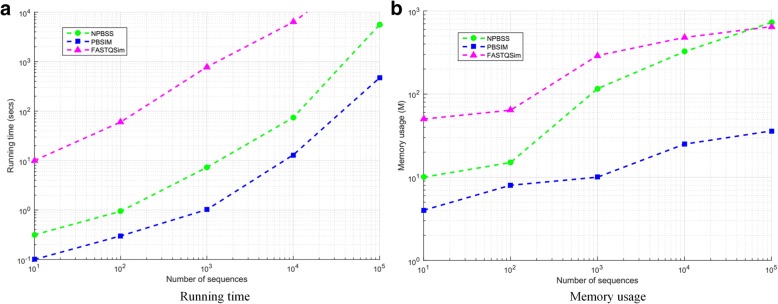
Running time (**a**) and memory usage (**b**) of NSSPB, PBSIM and FASTQSim with sequence number ranging from 10^2^ to 10^5^. A Linux machine with 2.40 GHz Intel Core i7 CPU (one core) was used. The MATLAB version of NPBSS was used in (**a**) and (**b**)

To test the computational complexity of NPBSS, *E. coli K12* genome was used as the reference sequence, and we simulated PacBio CLR reads from 10^2^ to 10^5^ reads number with an average sequence length of 8500. Here we report the computational time and memory requirements for CLR reads simulated by NPBSS, PBSIM and FASTQSim in Fig. 3, from which we can see that with the sequence number increases, the speed of NPBSS is lower than PBSIM, but faster than FASTQSim. The memory usage of NPBSS and FASTQSim is larger than PBSIM, and the memory requirement of NSSPB is a little larger than FASTQSim when sequence number increases to 10^5^.

### Assembly test for simulated reads

Finally, we conducted several assembly tests on the datasets simulated by NPBSS and PBSIM. Canu [30] tool is specifically designed for single-molecule sequences, and it continues to improve with increasing PacBio sequencing depth, reaching its maximum assembly continuity around 50X (depth). Thus, Canu tool was used to get the assembly results. We simulated PacBio CLR reads with NPBSS and PBSIM by fixing the sequencing depth as 5, 10, 15, 20, 25, 30, 35, 40, 45 and 50 for each reference genome, and setting the parameters of accuracy identically with the raw data.

**Fig. 4:**
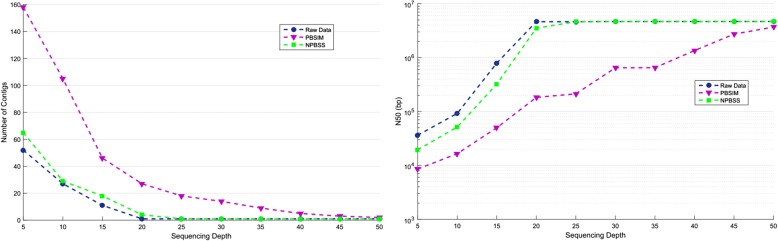
The numbers (left figure) and N50 (right figure) of contigs in the assembly test for *E.coli K12* data

The assembly results (i.e., contigs number, N50) for *E.coli K12* are shown in Fig. 4. N50 is the contig length such that using equal or longer contigs produces half the bases of the genome. From Fig. 4, we can see that with the depth increase, the number of contigs of raw data, PBSIM and NPBSS becomes smaller, while NPBSS obtained less contigs than PBSIM at each depth. It is evidently observed that the contigs number of NPBSS is much closer to the raw data than that of PBSIM. In addition, the N50 also shows that NPBSS gained similar contig length to the raw PacBio data, and the contig lengths of NPBSS are longer than that of PBSIM. These results show that the sequence simulation system of NPBSS is more realistic to real PacBio CLR data than PBSIM. Similar assembly results can be found in Additional file 1: Figures S6-S8 for genomes of *A. thaliana*, *D. melanogaster* and *C. elegans*, respectively.

### Extensibility of NPBSS

In order to test the reliability and generalization of NPBSS for a new PacBio sequencing data, we simulated PacBio CLR reads using *Neurospora crassa* genome, a fungus organism. The raw PacBio CLR dataset of *N. crassa* can be download from PacBio DevNet (https://github.com/PacificBiosciences/DevNet/wiki/Datasets).

**Fig. 5:**
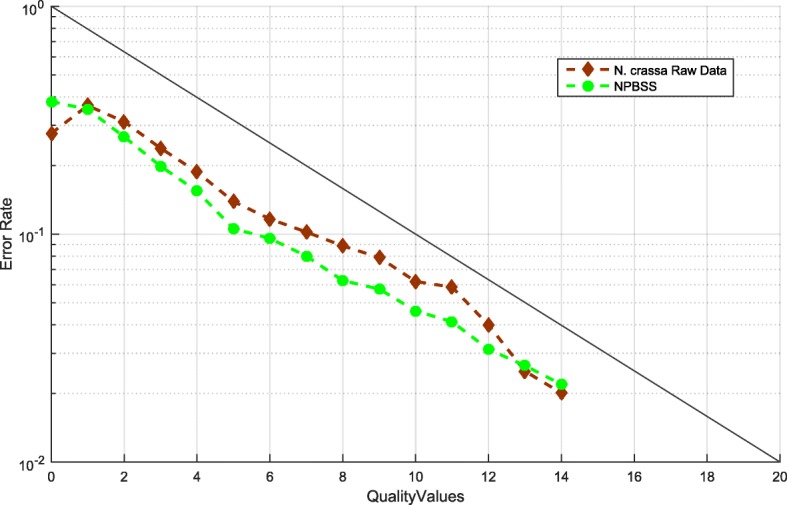
The relationship between error rate and QVs in simulated CLR reads generated by NPBSS for *N. crassa* genome

We generated PacBio CLR reads using NPBSS, and aligned the simulated reads to *N. crassa* reference genome to obtain different error rates. Table 1 reports the alignment results, from which we can see that the error rate of insertion, deletion and substitution are consistent with the raw data. Figure 5 represents the curve between error rate and different QVs, which is close to the raw data. These results show that NBPSS has a reliable extensibility to generate PacBio CLR reads for a new reference genome. We further used the *Homo sapiens* genome to test the reliability and generalization of NPBSS. The raw PacBio CLR dataset of *H. sapiens* can be download from http://datasets.pacb.com/2013/Human10x/READS/index.html. Additional file 1: Figure S9 describes the curve between error rate and different QVs for NPBSS, which shows the similar result in Fig. 5.

**Table 1:** Statistics of the simulated reads with NPBSS for genome of *N. crassa*

Methods	Match rate (%)	Insertion rate (%)	Deletion rate (%)	Substitution rate (%)	Total error rate (%)	Average length (bp)
Raw Data	83.354	2.878	8.758	5.010	16.646	5812
NPBSS	83.516	2.934	8.497	5.103	16.534	5889

### NPBSS for CCS reads generating

**Fig. 6:**
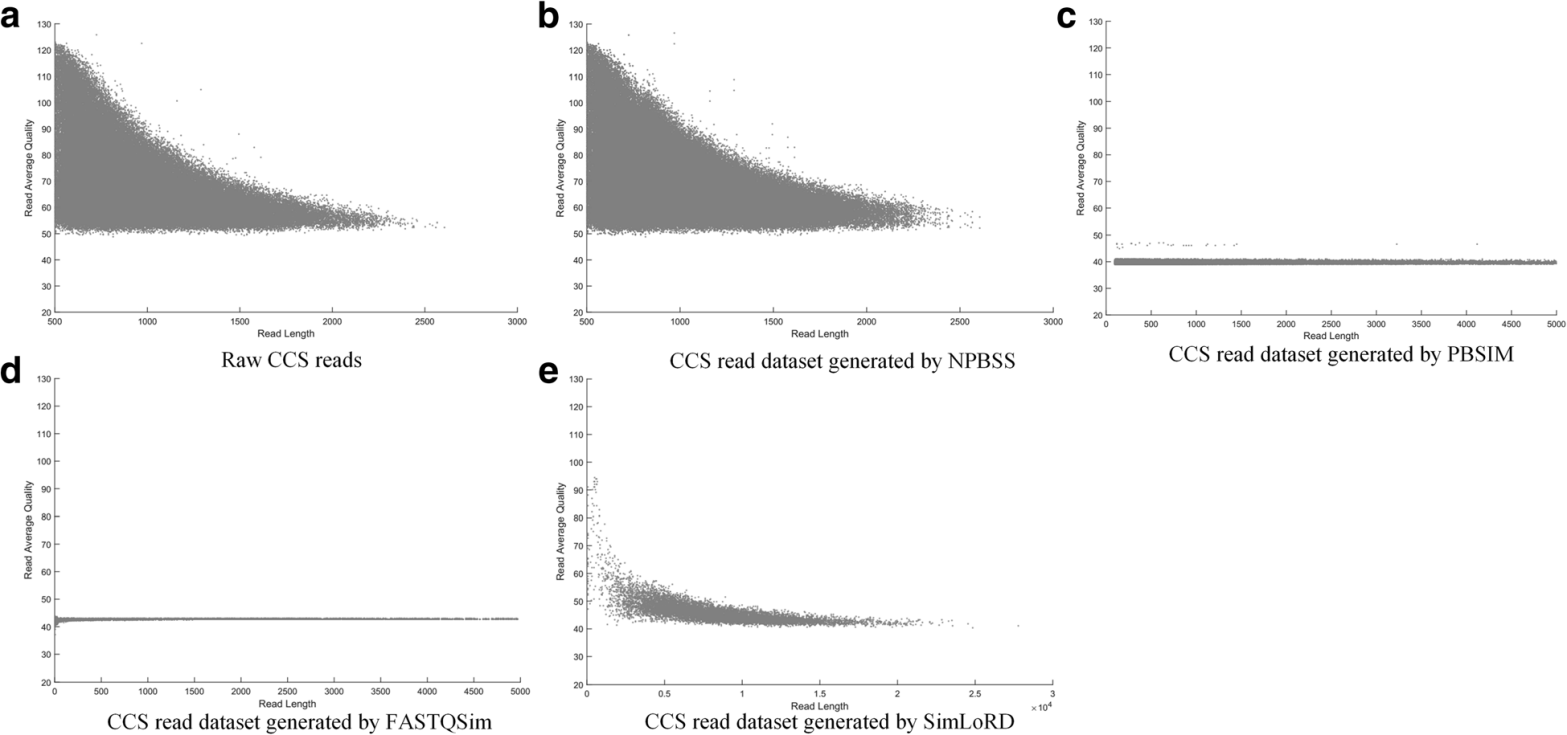
The scatter plot of read length and average base quality per read for CCS read datasets: (**a**) the raw CCS read dataset of *E. coli* K12 MG1655, (**b**) NPBSS simulation, (**c**) PBSIM simulation, (**d**) FASTQSim simulation and (**e**) SimLoRD simulation

Additionally, NPBSS can also generate PacBio CCS reads by using a sampling-based simulation (see Section 1 in Additional file 1). Two PacBio CCS datasets (*E. coli* K12 MG1655 and *E. coli* C227–11) in Additional file 1: Table S4 were applied to compare the simulation results. Figure 6 shows the scatter plot of read length and average base quality per read on *E. coli* K12 MG1655 raw dataset, and four CCS read datasets generated by NPBSS, FASTQSim, PBSIM and SimLoRD. It can be seen that NPBSS provides more realistic simulation results than FASTQSim and PBSIM. The output sequences of SimLoRD tool shown in Fig. 6e are the raw subreads with high errors, not the final corrected CCS reads with high accuracy. Similar simulation results can be found for *E. coli* C227–11 in Additional file 1: Figure S10.

**Fig. 7:**
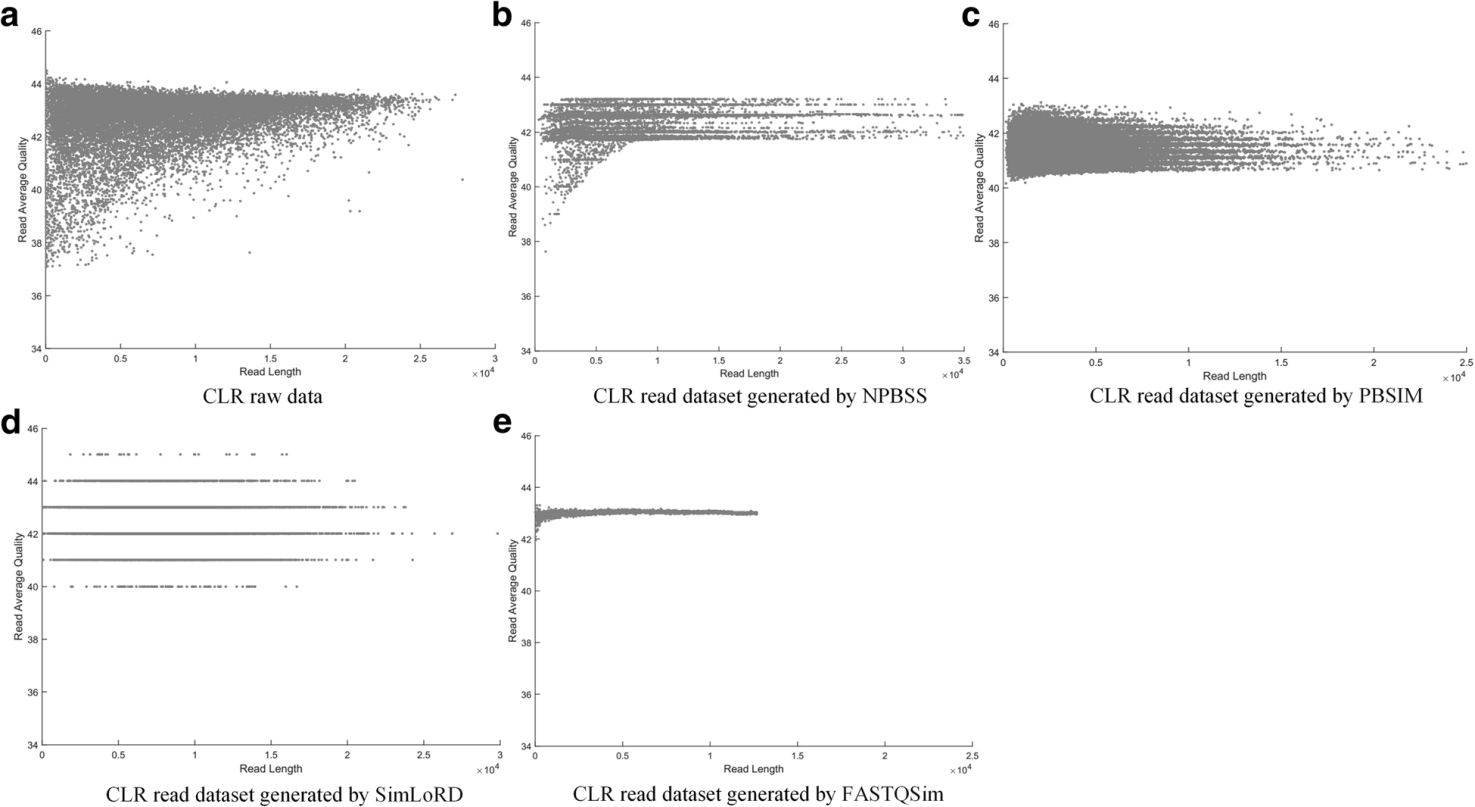
The scatter plot of read length and average base quality per read for CLR reads of *E.coli K12*: (**a**) the raw CLR data of *E.coli K12*; (**b**) NPBSS simulation; (**c**) PBSIM simulation; (**d**) SimLoRD simulation; and (**e**) FASTQSim simulation

Although SimLoRD tool is specialized for CCS reads, the simulation of CLR reads is also possible through setting the maximum number of passes to 1 and choosing the base error probabilities for substitution, deletion and insertion accordingly. And Fig. 7 shows the scatter plot of CLR read length and average base quality per read on *E. coli K12* raw dataset and four CLR read datasets generated by NPBSS, FASTQSim, PBSIM and SimLoRD. It can be seen that NPBSS provides more realistic simulation results than other tools. Similar simulation results can be found in Additional file 1: Figures S11-S13 for genomes of *A. thaliana*, *C. elegans* and *D. melanogaster*, respectively.

## Conclusions

The SMRT sequencing technology, developed by PacBio, has been widely used in the resequencing and de novo assembly studies. And more and more relevant computational applications have been developed for sequence analysis tasks from SMRT data, such as genome assembly, SNP calling and structural variant discovery. It becomes essential that these methods need to be benchmarked against other similar tools to show their superiority at least in some certain aspects. A genome sequencing simulation system can be very helpful for development and evaluation of these analysis tools. In addition, since no gold standard is available for sequencing data analysis, performance evaluation based on simulated sequencing is still the most effective way. Therefore, PacBio reads simulator becomes essential for facilitating the improvement of metagenomic tools and planning metagenomic projects. Although some simulators (e.g., PBSIM, FASTQSim and SimLoRD) targeted the PacBio platform have been proposed, neither of them considers the relationship between error rate and QVs. In this article, we designed and implemented an effective sequence simulator (NPBSS) for generating PacBio reads that are more like real PacBio data. NPBSS firstly samples the read sequences according to the read length logarithmic normal distribution, and choses different base QVs with different proportions. Then, NPBSS computes the overall error probability of each base in the read sequence with an empirical model, and calculates the deletion, substitution and insertion probabilities with the overall error probability to generate the PacBio sequences. The main advantage of NPBSS tool is that NPBSS applies an empirical model to capture the relationship between the error rate and QVs. Compared with existing PacBio reads simulators, alignment results demonstrate that NPBSS can fit the error rate of PacBio sequence data better. In addition, assembly tests on the simulated sequences of NPBSS also show that the number and length of contigs are more like real PacBio datasets. NPBSS can be very helpful to develop and evaluate subsequent analysis tools based on PacBio sequencing.

## Methods

NPBSS’s processing pipeline mainly consists of the following four phases: 1) Modeling the length of CLR and CCS reads according to the logarithmic normal distribution; 2) Selecting the different QVs based on the different proportions; 3) Calculating an overall error probability of each position based on the empirical model; and 4) Obtaining the deletion, substitution and insertion probabilities based on the overall error probability. A detail description for each parameter setting of NPBSS is presented in Additional file 1: Table S11, which will be convenient for usage.

1. Modeling the length distribution

According to observed distributions of read length in (Additional file 1 Figures S1-S2 ), the logarithmic normal distribution (Eq.1) was used to model the length of CLR reads.

1 $$ p\left(x;\mu, \sigma \right)=\frac{1}{x\sigma \sqrt{2\pi }}\kern0.5em \exp \kern0.62em \left[-\kern0.5em \frac{{\left(\kern0.5em In\kern0.62em x\kern0.5em -\kern0.5em \mu \kern0.5em \right)}^{\kern0.5em 2}}{2\kern0.5em {\sigma}^2}\right] $$

where variable *x* is the read length.*μ*and*σ*are the mean value and standard deviation of the variable *x* natural logarithm, which can be estimated with the observed reads length (see Additional file 1 Section 1).

2) Selecting QV

QVs measure the probability that a base is sequenced incorrectly, revealing the error probability of each base. In order to find the proportions of different QVs, we counted the number of each QV for every real CLR datasets, providing the proportion of different QVs of the four CLR datasets in Additional file 1: Figure S14 and Table S12. Then, NPBSS will select different QVs according to the average proportion in Additional file 1: Figure S14 for each read.

3) Error model

Theoretically, the QV of each base in read sequence is logarithmically related to the base error probability *P*_*th*_, that is, the *P*_*th*_ value of each base can be calculated by:

2 $$ {P}_{th}={10}^{-\kern0.5em \frac{QV}{10}} $$

In fact, the actual error probability *P*_*error*_ is lower than the theoretical value *P*_*th*_. In order to obtain the actual *P*_*error*_, we first took the four CLR raw datasets analyzed in Additional file 1: Table S4 to get the relationship (see Additional file 1: Figure S15 and Table S13) between error rate and QVs hidden in PacBio sequences. Then, by using the least square method, we found the following Eq. 3 of fitting curve (i.e., thick dark-gray line in Additional file 1: Figure S15) to estimate the actual overall error probability of each base in sequences.

3 $$ {P}_{error}=0.3942\ast {10}^{-\frac{QV}{10}}+0.0041 $$

where *P*_*error*_ is the actual overall error probability when a QV is given. This model is more consistent with the error rate of real PacBio sequencing data. The *P*_*error*_ value of each QV is shown in Additional file 1: Table S14

4) Deletion, substitution and insertion errors

After getting the overall error probability (*P*_*error*_) of each position base, the deletion, substitution and insertion probabilities can be calculated by the Eq. 3–5 (in Additional file 1 Section 1).

## Availability and requirements

**Project name**: NPBSS

**Project home page**:

Octave version: https://github.com/NWPU-903PR/NPBSS_Octave

MATLAB version: https://github.com/NWPU-903PR/NPBSS_MATLAB

**Operating system(s)**: Windows

**Programming language**: MATLAB and Octave

Other requirements: MATLAB and Octave Environment

License: GNU GPL v.3

Any restrictions to use by non-academics: None

## Additional file

Additional file 1: Supplementary Material (including supplementary figures and tables) for NPBSS. (PDF 2331 kb)

## Abbreviations

CCS: Circular consensus sequencing
CLR: Continuous long reads
PacBio: Pacific Biosciences
QV: Quality value (the Phred quality score)
SMRT: Single molecule, real-time;
