# Supplementary material for: NPBSS: a new PacBio sequencing simulator for generating the continuous long reads with an empirical model
Source: BMC Bioinformatics. 2018 May 22;19:177. doi: 10.1186/s12859-018-2208-0 (PMC5964698; doi:10.1186/s12859-018-2208-0)
Supplement: Supplementary file 1 — Supplementary Material (including supplementary figures and tables) for NPBSS. (PDF 2331 kb) [file 12859_2018_2208_MOESM1_ESM.pdf]

NPBSS: A new PacBio sequencing simulator for generating the continuous long reads with an empirical model

Ze-Gang Wei, Shao-Wu Zhang*

Key Laboratory of Information Fusion Technology of Ministry of Education, College of Automation, Northwestern Polytechnical University, Xi'an, 710072, China

* Corresponding author. Email: zhangsw@nwpu.edu.cn.

Section 1. NPBSS Methods

Modeling the length distribution

Suppose that variable x indicates the read length, its probability density function can be expressed as [1]:

$$p(x;\mu,\sigma)=\frac{1}{x\sigma\sqrt{2\pi}}\exp\left[-\frac{(In\,x-\mu)^2}{2\sigma^2}\right] \tag{1}$$

where μ and σ are the mean value and standard deviation of the variable x natural logarithm. If we use $E(x)$ and $Var(x)$ to respectively represent the mean value and variance of the read length derived from Fig.S1-S2, the parameters μ and σ can be estimated by the following equations.

$$\begin{cases} E(x)=\exp\left(\mu+\frac{\sigma^2}{2}\right) \\ Var(x)=\left[\exp(\sigma^2)-1\right]\exp(2\mu+\sigma^2) \end{cases} \tag{2}$$

Thus, according to observed distributions of read length, the logarithmic normal distribution (Eq.1) can be used to model the length of CLR reads. Or, after the users provide the parameters of average read length and variance, NPBSS simulates the distribution of read length by using the Eq.1.

Deletion, substitution and insertion errors assignment

After getting the overall error probability (P_{error}) of each base in read sequence, the deletion, substitution and insertion probabilities can be calculated by the following equations:

$$P_{del} = P_{error} \times \frac{R_{del}}{R_{del} + R_{ins} + R_{sub}} \quad (3)$$

$$P_{ins} = P_{error} \times \frac{R_{ins}}{R_{del} + R_{ins} + R_{sub}} \quad (4)$$

$$P_{sub} = P_{error} \times \frac{R_{sub}}{R_{del} + R_{ins} + R_{sub}} \quad (5)$$

where P_{del}, P_{ins} and P_{sub} are the probabilities of deletion, insertion and substitution of base in read sequence, respectively. R_{del}, R_{ins} and R_{sub} are the average accuracy over the length of each read, which can be taken from a normal distribution with parameters given by user, that is: $R_{del} \sim N(\mu_{del}, \sigma_{del})$, $R_{ins} \sim N(\mu_{ins}, \sigma_{ins})$ and $R_{sub} \sim N(\mu_{sub}, \sigma_{sub})$. The mean values and standard deviation also can be changed by users, which is more flexible to simulate.

NPBSS for CCS generation

For PacBio CCS reads simulation, NPBSS adopted a sampling-based simulation. In the CCS simulation, lengths and quality scores of reads are simulated by randomly sampling them in a real library of PacBio CCS reads (provided by the user). Subsequently, different errors of each base in read sequences are simulated by the same method with the CLR model-based simulation.

Section 2. Figures and Tables

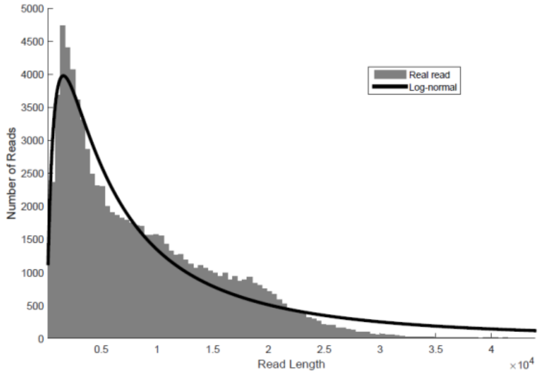

(a) *E. coli K12*

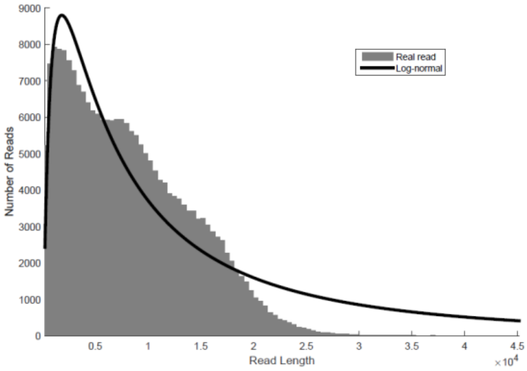

(b) *A. thaliana*

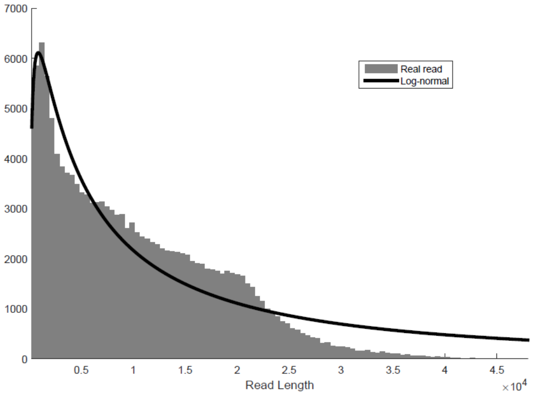

(c) *C. elegans*

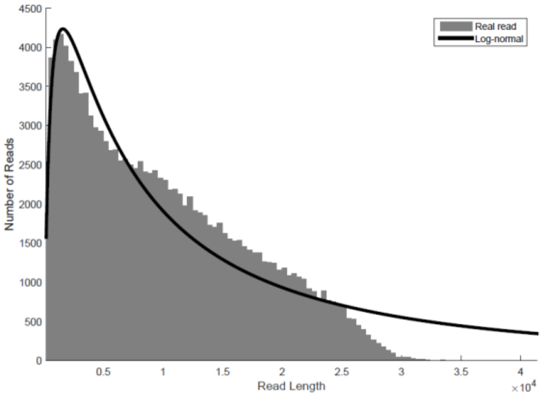

(d) *D. melanogaster*

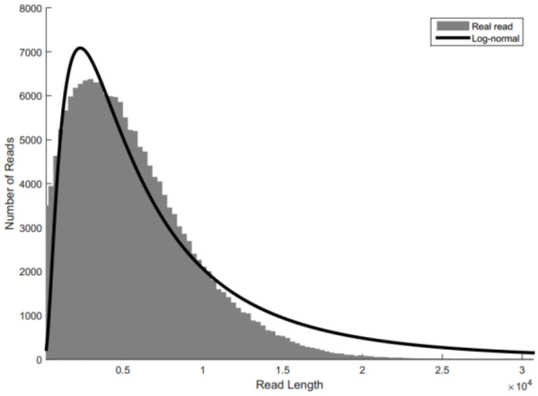

(e) *N. crassa*

Fig. S1 Distributions of CLR lengths of four datasets generated by PacBio RS platform. The column diagram and black lines indicate the distribution of lengths of real reads and a log-normal distribution, respectively.

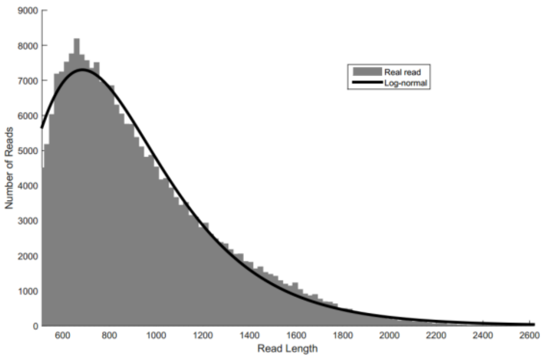

(a) *E. coli* K12 MG1655

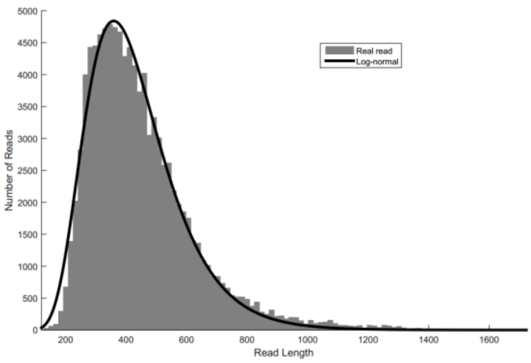

(b) *E. coli* C227-11

Fig. S2 Distributions of CCS lengths of two datasets generated by PacBio platform. The column diagram and black lines indicate the distribution of lengths of real reads and a log-normal distribution, respectively.

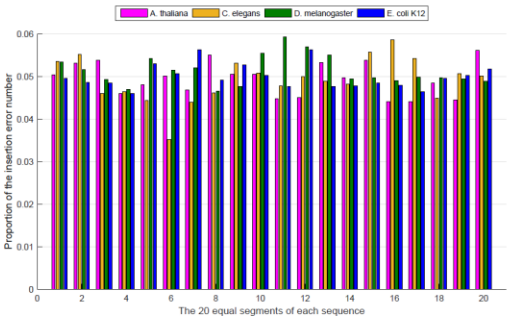

(a)

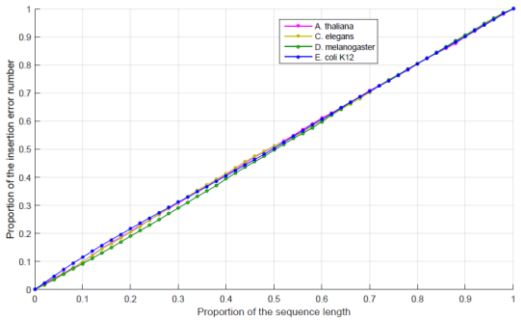

(b)

Fig. S3 Insertion error distribution. (a) The distribution of insertion errors in the equally divided sequence segments intervals. (b) The corresponding cumulative curves between the sequence length and number of errors.

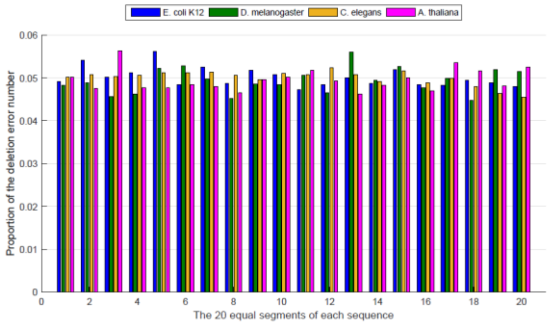

(a)

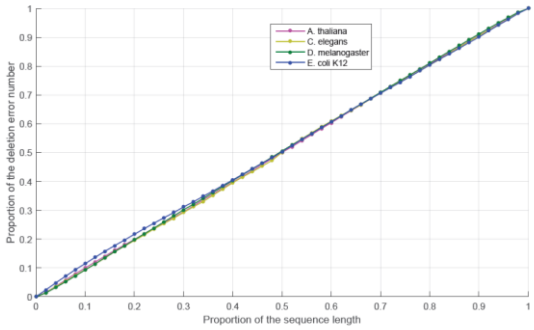

(b)

Fig. S4 Deletion error distribution. (a) The average distribution of deletion errors in the equal divided sequence segments intervals. (b) The corresponding cumulative curves between the sequence length and number of errors.

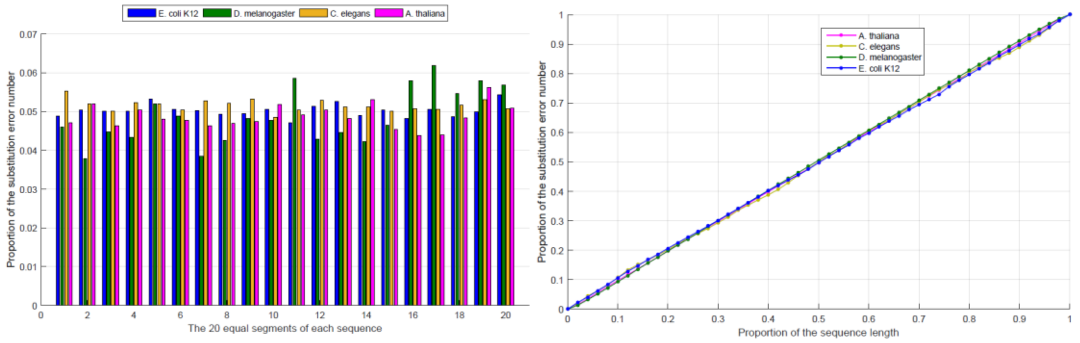

(a) (b)

Fig. S5 Substitution error distribution. (a) The average distribution of substitution errors in the equal divided sequence segments intervals. (b) The corresponding cumulative curves between the sequence length and number of errors.

Remarks for Fig. S3-S5

- Because the length of PacBio reads varies greatly (from hundreds bp to thousands bp), we equally divided one aligned sequence into 20 fragments, then separately counted the number of errors of insertion, deletion and substitution in each fragment. Thus, the proportion of error number can be defined as that the error number in each segment is divided by the total number (the sum number of errors in all fragments) of the whole sequence.
- It is obvious that the error distribution of insertion, deletion and substitution present us a nice uniform layout, therefore, the errors of PacBio differ with the second-generation sequencing systems that are concentrated around certain segments (e.g., errors increase in distal segment and do not occur randomly) [2]. The PacBio sequencing errors occur randomly in one sequence of the PacBio sequencing system.

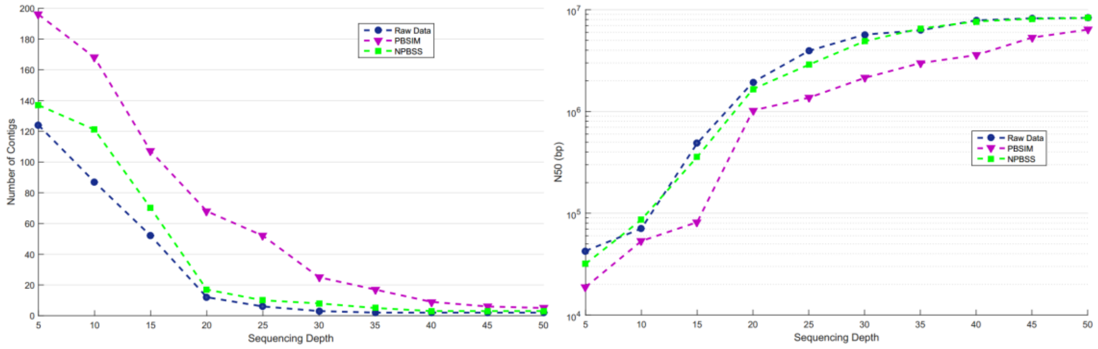

Fig. S6 The numbers (left figure) and N50 (right figure) of contigs in the assembly test for *A. thaliana* data.

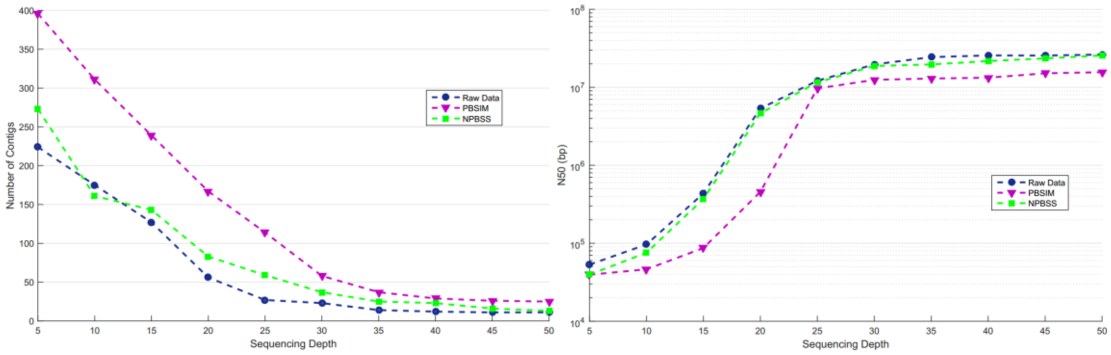

Fig. S7 The numbers (left figure) and N50 (right figure) of contigs in the assembly test for *D. melanogaster* data.

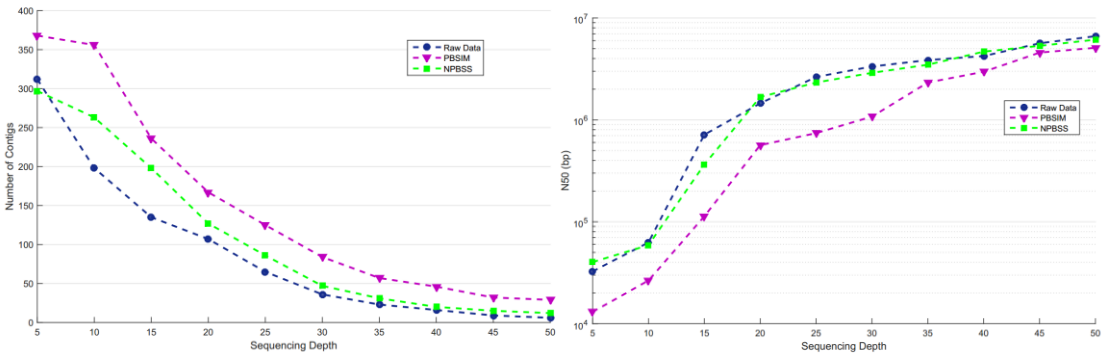

Fig. S8 The numbers (left figure) and N50 (right figure) of contigs in the assembly test for *C. elegans* data.

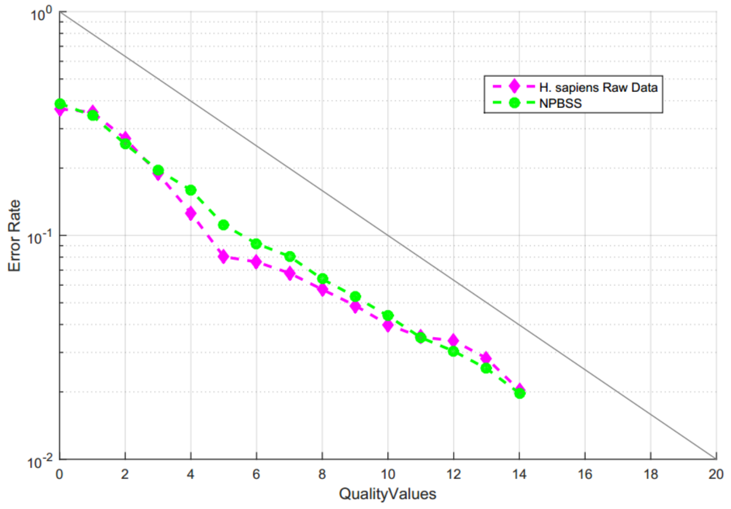

Fig. S9 The relationship between error rate and QVs in simulated CLR reads generated by NPBSS for *H. sapiens* genome.

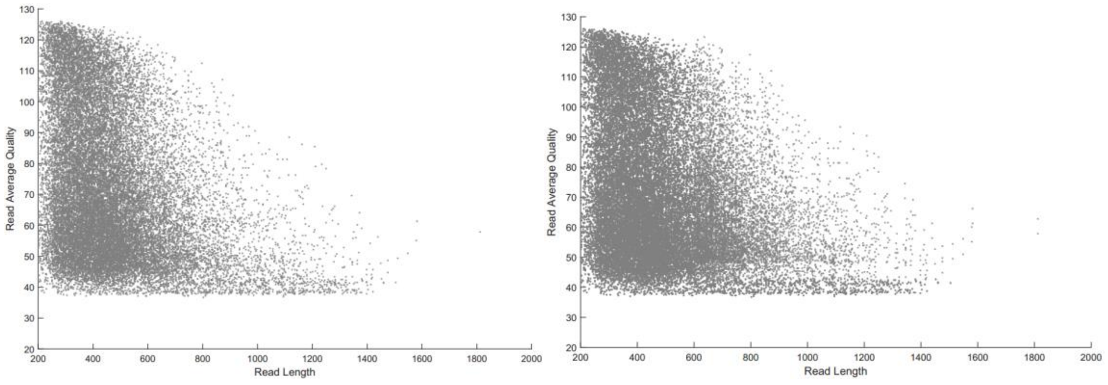

(a) Raw CCS data

(b) CCS read dataset generated by NPBSS

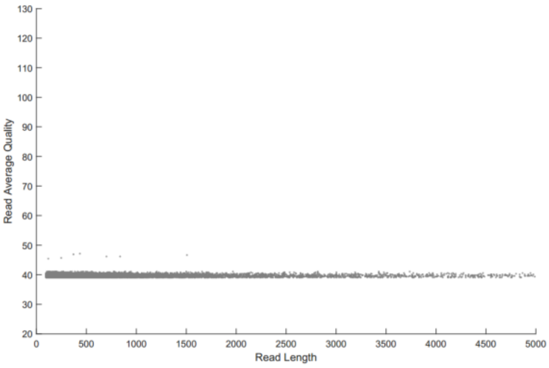

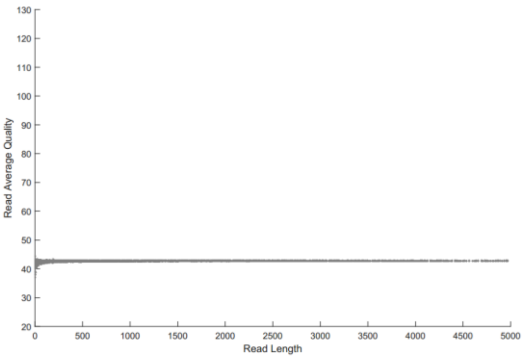

(c) CCS read dataset generated by PBSIM (d) CCS read dataset generated by FASTQSim

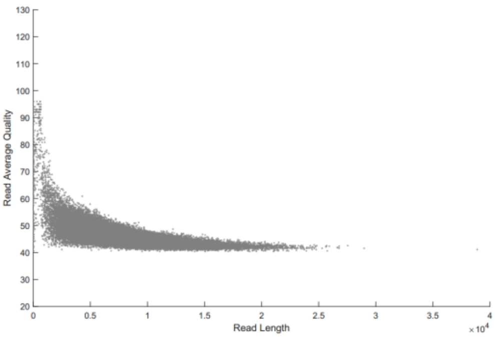

(e) CCS read dataset generated by SimLoRD

Fig. S10 The scatter plot of read length and average base quality per read for CCS reads: (a) the raw CCS data of *E. coli* C227-11; (b) NPBSS simulation; (c) PBSIM simulation; (d) FASTQSim simulation; and (e) SimLoRD simulation.

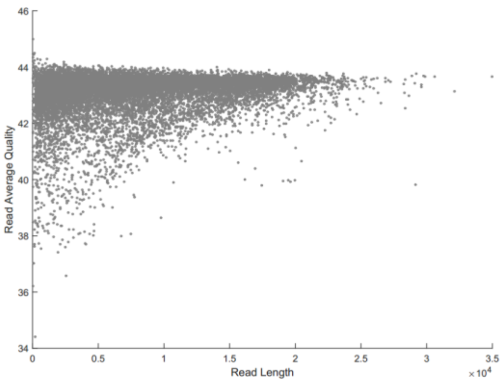

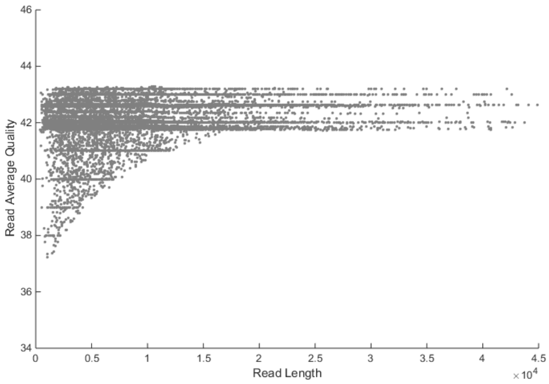

(a) CLR raw data (b) CLR read dataset generated by NPBSS

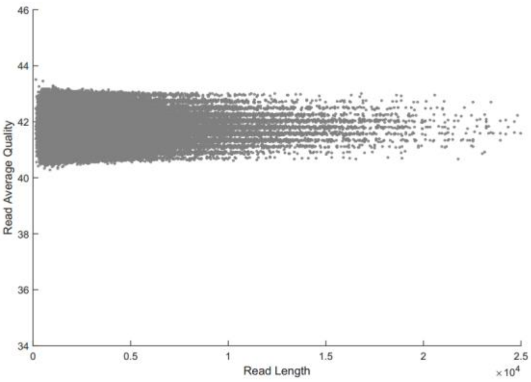

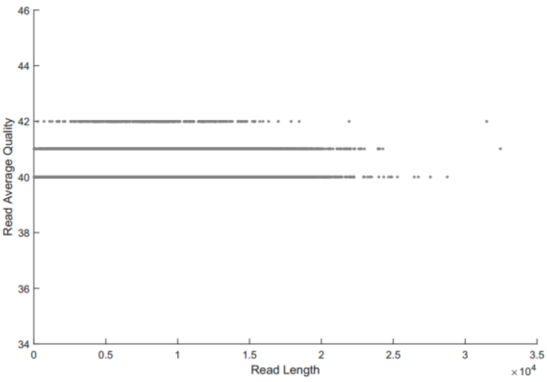

(c) CLR read dataset generated by PBSIM

(d) CLR read dataset generated by SimLoRD

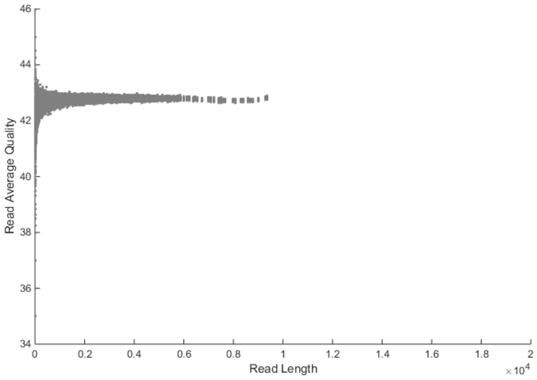

(e) CLR read dataset generated by FASTQSim

Fig. S11 The scatter plot of read length and average base quality per read for CLR reads of *A. thaliana*: (a) the raw CLR data of *A. thaliana*; (b) NPBSS simulation; (c) PBSIM simulation; (d) SimLoRD simulation; and (e) FASTQSim simulation.

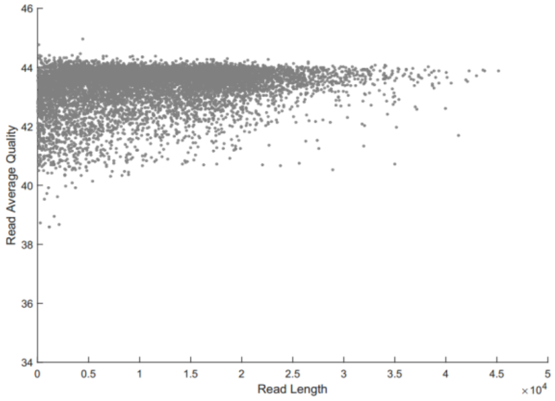

(a) CLR raw data

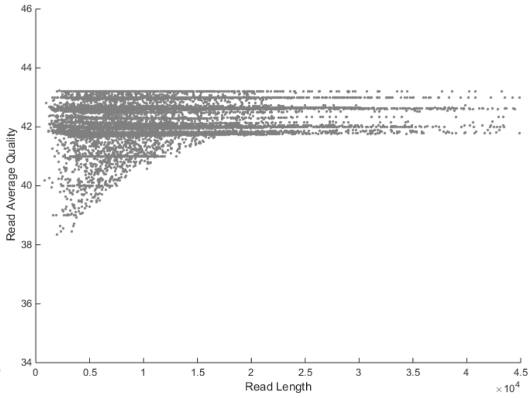

(b) CLR read dataset generated by NPBSS

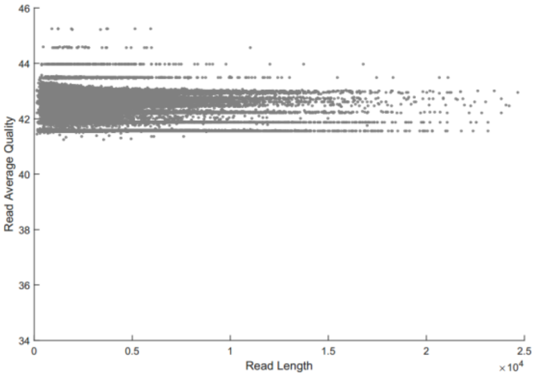

(c) CLR read dataset generated by PBSIM

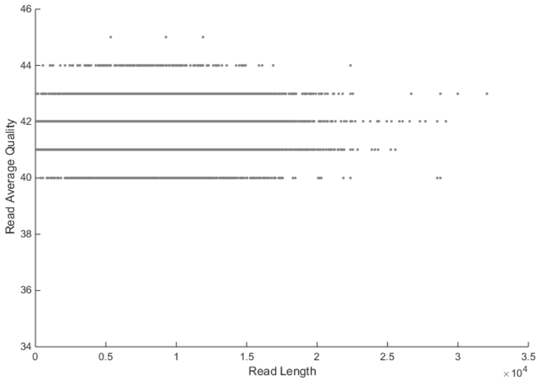

(d) CLR read dataset generated by SimLoRD

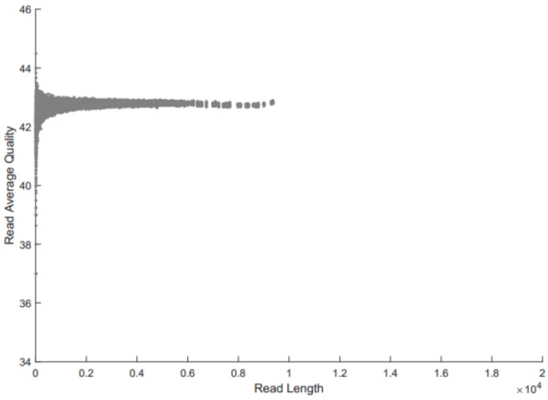

(e) CLR read dataset generated by FASTQSim

Fig. S12 The scatter plot of read length and average base quality per read for CLR reads of *C. elegans*: (a) the raw CLR data of *C. elegans*; (b) NPBSS simulation; (c) PBSIM simulation; (d) SimLoRD simulation; and (e) FASTQSim simulation.

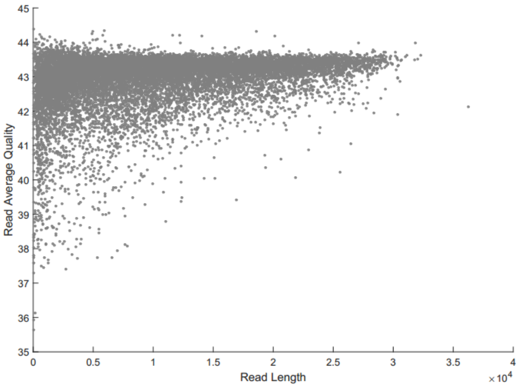

(a) CLR raw data

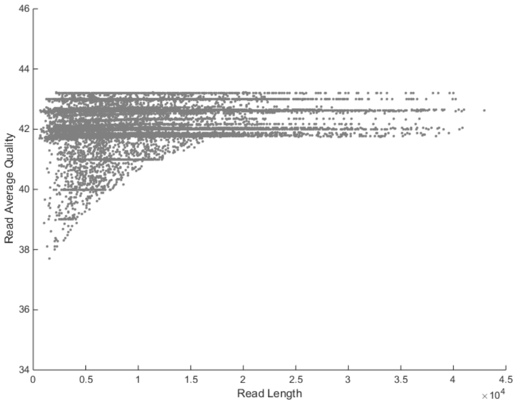

(b) CLR read dataset generated by NPBSS

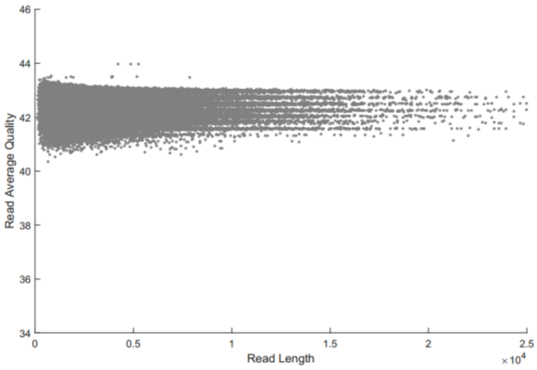

(c) CLR read dataset generated by PBSIM

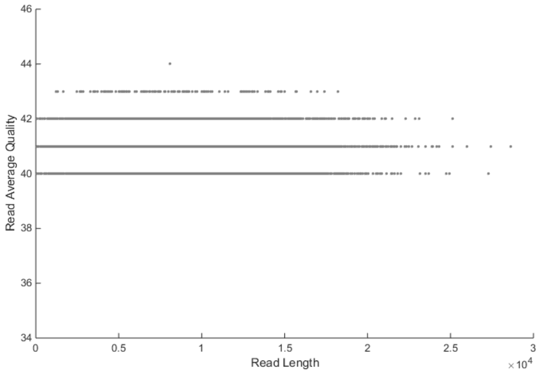

(d) CLR read dataset generated by SimLoRD

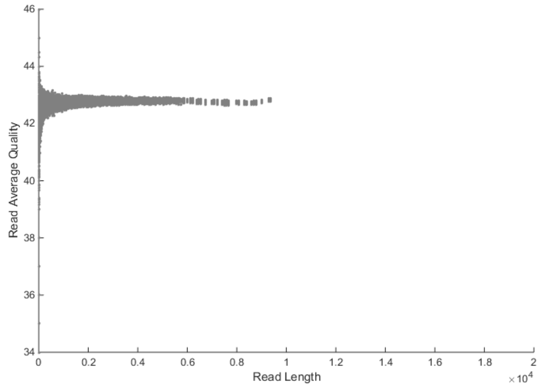

(e) CLR read dataset generated by FASTQSim

Fig. S13 The scatter plot of read length and average base quality per read for CLR reads of *D. melanogaster*: (a) the raw CLR data of *D. melanogaster*; (b) NPBSS simulation; (c) PBSIM simulation; (d) SimLoRD simulation; and (e) FASTQSim simulation.

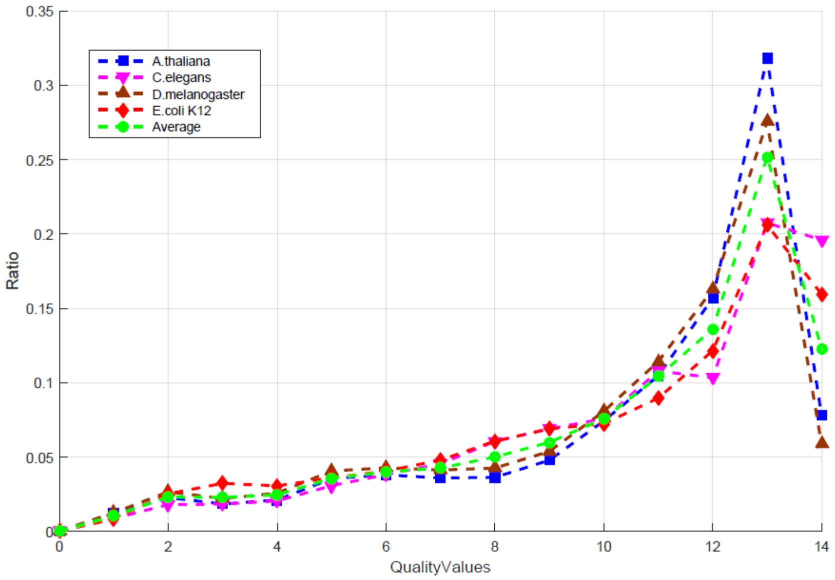

Fig. S14 The proportion of different QVs in the four CLR datasets of *E. coli K12*, *C. elegans*, *A. thaliana* and *D. melanogaster*. We can see that the proportion curves present a rising trend with the QV growth from 0 to 13, and all of them have a peak value at QV=13 for each dataset, then drop down when QV=14. NPBSS will select different QVs according to the average proportion (green line).

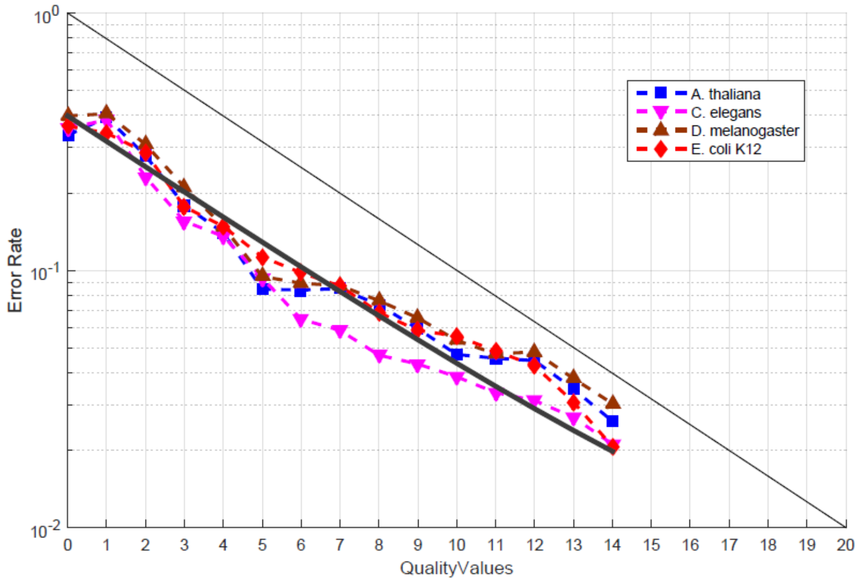

Fig. S15 The curve between actual error rate and QVs in the four CLR datasets of *E. coli K12*, *C. elegans*, *A. thaliana* and *D. melanogaster*. We can see that the error rate curves are lower than the diagonal and present a downtrend with the increase of QV, meaning that the larger the QV, the lower the probability of error. These results show that the QV has a strong relation to the average error rate for each PacBio sequence, which should be considered when simulating PacBio sequences. The thick dark-gray line is the fitting curve produced by using the least square method.

Table S1. PacBio datasets website links

Datasets		Website links
CLR	*E. coli K12*	https://github.com/PacificBiosciences/DevNet/wiki/E.-coli-Bacterial-Assembly
	C. elegans	https://github.com/PacificBiosciences/DevNet/wiki/C.-elegans-data-set
	A. thaliana	https://github.com/PacificBiosciences/DevNet/wiki/Arabidopsis-P5C3
	D. melanogaster	https://github.com/PacificBiosciences/DevNet/wiki/Datasets
CCS	*E. coli* K12 MG1655	https://github.com/PacificBiosciences/DevNet/wiki/
	E. coli C227-11	https://www.ncbi.nlm.nih.gov/sra/SRX081567

Table S2. Assembly genome website links

Genome	Website links
E. coli K12	https://s3.amazonaws.com/files.pacb.com/datasets/secondary-analysis/e-coli-k12-P6C4/polished_assembly.fastq.gz
C. elegans	http://datasets.pacb.com.s3.amazonaws.com/2014/c_elegans/40X/polished_assembly/polished_assembly.fasta.gz
A. thaliana	http://datasets.pacb.com.s3.amazonaws.com/2014/Arabidopsis/reads/polished_assembly.fasta
D. melanogaster	https://s3.amazonaws.com/datasets.pacb.com/2014/Drosophila/reads/dmel_FALCON_diploid_assembly.tgz

Table S3. Statistics of four real CLR datasets

Datasets	*E. coli K12*	*C. elegans*	*A. thaliana*	*D. melanogaster*
Enzyme-chemistry	P6-C4	P6-C4	P5-C3	P5-C3
Total number bases	748,508,257 bp	4,120,796,736 bp	1,769,374,127 bp	15,208,567,933 bp
Coverage depth	160	40	65	106
Number of reads	87,217	383,544	215,026	1,514,730
Read length				
Average	8,582	10,744	8,229	10,040
Maximum	44,113	48,211	45,400	41,579
Top 5% of reads	>21 kb	>24 kb	>18 kb	>23 kb
Half of data in reads	>6 kb	>9 kb	>7 kb	>6 kb

Remark: It can be seen from Table S3 that the current PacBio sequencer can boast average read lengths over 10 kb (for both *C. elegans* and *D. melanogaster* datasets), with an N50 of more than 6 kb (that is, over half of all data are in reads longer than 6 kb), which is drastically longer than the maximum read length (only paired-end 250 bp) generated by Illumina HiSeq 2500 [3].

Table S4. Statistics of two PacBio CCS datasets

**Datasets**	***E. coli* K12 MG1655**	***E. coli* C227-11**
Total number bases	217,871,193 bp	223,962,022 bp
Depth	45	41
Number of reads	231,629	502,157
Read length		
Average	940	446
Standard deviation	332	168
Maximum	2,627	1,864
Minimum	200	116

Table S5. The proportions of different type errors in these four CLR raw datasets (%)

**Type of data**	**Type of errors**	**Maximum**	**Minimum**	**Average**	**Standard deviation**
E. coli K12	Match	92.971	73.770	88.345	3.089
	Substitution	17.656	0.278	2.300	1.857
	Insert	18.674	0.304	2.953	2.120
	Deletion	21.368	1.483	6.403	2.761
C. elegans	Match	91.871	83.364	86.543	1.325
	Substitution	9.150	1.607	6.326	0.940
	Insert	5.777	0.273	2.839	1.321
	Deletion	7.227	1.669	4.292	1.127
A. thaliana	Match	93.097	71.719	80.051	2.960
	Substitution	21.811	0.913	11.559	2.912
	Insert	11.748	0.348	2.525	1.034
	Deletion	17.822	2.080	5.865	1.521
D. melanogaster	Match	93.449	71.296	83.147	4.896
	Substitution	19.644	0.268	6.907	4.080
	Insert	12.599	0.172	3.133	1.633
	Deletion	17.936	1.195	6.813	1.930

From Table S5 it can be seen that although the accuracy of different datasets varies, the match accuracy is in the range of 83% to 88%, which is near to the error rate PacBio reported [4].

Table S6. The proportions of different type errors in these two CCS raw datasets (%)

**Type of data**	**Type of errors**	**Maximum**	**Minimum**	**Average**	**Standard deviation**
***E. coli* K12 MG1655**	Match	100.00	73.312	97.730	2.538
	Substitution	11.978	0.00	3.740	1.585
	Insert	9.317	0.00	0.939	1.165
	Deletion	10.777	0.00	0.957	1.084
***E. coli* C227-11**	Match	100.00	76.145	98.231	2.417
	Substitution	10.624	0.00	0.808	1.406
	Insert	8.148	0.00	1.034	1.154
	Deletion	9.567	0.00	0.456	1.071

Table S7 Statistics of the simulated reads with PBSIM, FASTQSim and NPBSS for genome of *E. coli K12*

Methods	Match rate (%)	Insertion rate (%)	Deletion rate (%)	Substitution rate (%)	Total error rate (%)	Average length (bp)
Raw Data	88.345	2.953	6.403	2.300	11.656	8,517
PBSIM	87.031	2.751	6.878	2.652	12.281	8,826
FASTQSim	89.117	2.614	6.028	2.246	10.915	8,421
NPBSS	88.976	2.809	6.563	2.437	11.809	8,596

Table S8 Statistics of the simulated reads with PBSIM, FASTQSim and NPBSS for genome of *C. elegans*

Methods	Match rate (%)	Insertion rate (%)	Deletion rate (%)	Substitution rate (%)	Total error rate (%)	Average length (bp)
Raw Data	86.543	2.839	4.292	6.326	13.457	10,132
PBSIM	86.162	2.893	4.712	7.047	14.652	10,547
FASTQSim	87.065	2.743	4.039	6.156	12.938	9,986
NPBSS	86.324	2.851	4.076	6.482	13.409	10,476

Table S9 Statistics of the simulated reads with PBSIM, FASTQSim and NPBSS for genome of *A. thaliana*

Methods	Match rate (%)	Insertion rate (%)	Deletion rate (%)	Substitution rate (%)	Total error rate (%)	Average length (bp)
Raw Data	80.051	2.525	5.865	11.559	19.949	8,241
PBSIM	79.347	2.612	6.149	11.661	20.422	8,309
FASTQSim	80.187	2.634	5.126	12.056	19.816	8,117
NPBSS	80.195	2.498	5.931	11.587	20.016	8,275

Table S10 Statistics of the simulated reads with PBSIM, FASTQSim and NPBSS for genome of *D. melanogaster*

Methods	Match rate (%)	Insertion rate (%)	Deletion rate (%)	Substitution rate (%)	Total error rate (%)	Average length (bp)
Raw Data	83.147	3.133	6.813	6.907	16.853	10,049
PBSIM	84.696	3.678	6.361	6.972	17.011	11,032
FASTQSim	84.925	3.016	6.124	5.941	15.081	9,978
NPBSS	83.172	3.652	6.308	6.836	16.796	10,592

Table S11. Optional arguments of NPBSS

Options	Description [default]
-dep	sequencing depth [20]
-n	number of reads to simulate
-min	minimum sequence length [100]
-max	maximum sequence length (default:45000).
-lg mean std	the mean and standard deviation for log-normal [8500 6930]
-len	average sequence length [8500]
-sub	substitution error rate between 0 to 1 [0.06]
-ins	insertion error rate between 0 to 1 [0.03]
-del	deletion error rate between 0 to 1 [0.06]
-samp	sample the read length from a FASTA file provided by users
-sam	create SAM format output [0]
-model	error model provided by users, not recommended to change
-qv	QVs selection table, not recommended to change

Table S12. The proportions of each quality value for CLR datasets

QVs	Proporation				Average
	E. coli K12	*A. thaliana*	*C. elegans*	*D. melanogaster*	
0	0.0002767	0.000201605	0.0001635	0.0002863	0.0002863
1	0.0083244	0.011920808	0.0087469	0.0125234	0.0125234
2	0.0253281	0.022706822	0.0180355	0.0265938	0.0265938
3	0.0323038	0.018465691	0.018373	0.0221681	0.0221681
4	0.0306581	0.021156354	0.0205046	0.025743	0.025743
5	0.0359439	0.035515384	0.0307281	0.0406372	0.0406372
6	0.0405824	0.037991929	0.038162	0.0427691	0.0427691
7	0.0479063	0.035942184	0.0454116	0.0412257	0.0412257
8	0.0606712	0.036334864	0.0604958	0.0426037	0.0426037
9	0.0689327	0.04788296	0.0690272	0.0537985	0.0537985
10	0.0724405	0.074258115	0.0757777	0.0807213	0.0807213
11	0.0895916	0.104679742	0.1078311	0.1141539	0.1141539
12	0.1214606	0.156821558	0.1033919	0.1626757	0.1626757
13	0.2060074	0.318315629	0.2074021	0.2754374	0.2754374
14	0.1595721	0.077806355	0.1959488	0.0586628	0.0586628

Table S13. The corresponding error rate values for CLR datasets

QVs	Theoretical Error Rate*	Actual Error Rate			
		E. coli K12	***A. thaliana***	***C. elegans***	***D. melanogaster***
0	1.0	0.363441	0.335061	0.356124	0.398278
1	0.794328	0.342079	0.392718	0.38506	0.406250
2	0.630957	0.286841	0.279143	0.230829	0.308902
3	0.501187	0.176615	0.178972	0.155073	0.210356
4	0.398107	0.148211	0.139980	0.135561	0.147830
5	0.316228	0.112745	0.084524	0.093556	0.094655
6	0.251189	0.097835	0.083806	0.064551	0.089055
7	0.199526	0.086878	0.084875	0.058410	0.087177
8	0.158489	0.068557	0.073402	0.046792	0.076213
9	0.125893	0.058395	0.059289	0.043206	0.065260
10	0.1	0.055463	0.047066	0.038546	0.053380
11	0.079433	0.048612	0.045522	0.033444	0.047343
12	0.063096	0.042561	0.044553	0.031186	0.048163
13	0.050119	0.030721	0.034546	0.026841	0.037958
14	0.039811	0.020678	0.025923	0.021077	0.030345

*The Theoretical Error Rate is computed by: $p=10^{-QV/10}$, where QV$\in$ [0,14]

Table S14. The actual overall error probability (P_{error}) of each QV.

QVs	0	1	2	3	4	5	6	7	8	9	10	11	12	13	14
P_{error}	0.398	0.317	0.253	0.202	0.161	0.129	0.103	0.083	0.067	0.054	0.044	0.035	0.029	0.024	0.020

Table S15. The characteristics of each simulator.

Simulators	Advantages	Limitations
PBSIM	Easy parameter settings.<br>Low computational complexity.<br>Generating both CLR and CCS sequences.	No SAM-formatted output.<br>Don't consider the relationship between. error rate and QVs.
FASTQSim	Platforms-independent (Illumina, 454, PacBio and IonTorrent).<br>For both read analysis and simulation.<br>Generating both CLR and CCS sequences.	High running time.<br>No SAM-formatted output.<br>Difficult to change parameters directly.<br>Don't consider the relationship between error rate and QVs.
SimLoRD	Easy parameter settings.<br>Specified for CCS sequence simulation.<br>Low computational complexity.	Normal performance for CLR reads.<br>Don't consider the relationship between error rate and QVs.
NPBSS	Consider the relationship between error rate and QVs.<br>Easy parameter settings.<br>Generating both CLR and CCS sequences.	Medium computational complexity.

References

1. Ono, Y., K. Asai, and M. Hamada, *PBSIM: PacBio reads simulator—toward accurate genome assembly.* Bioinformatics, 2012. **29**(1): p. 119-121.
2. Schirmer, M., et al., *Insight into biases and sequencing errors for amplicon sequencing with the Illumina MiSeq platform.* Nucleic acids research, 2015: p. gku1341.
3. Reuter, J.A., D.V. Spacek, and M.P. Snyder, *High-throughput sequencing technologies.* Molecular cell, 2015. **58**(4): p. 586-597.
4. Rhoads, A. and K.F. Au, *PacBio sequencing and its applications.* Genomics, proteomics & bioinformatics, 2015. **13**(5): p. 278-289.
